# Effect of Network Structure and Adaptive Foraging on Pollination Services of Species-Rich Plant-Pollinator Communities

**DOI:** 10.1093/icb/icaf024

**Published:** 2025-05-05

**Authors:** Fernanda S Valdovinos

**Affiliations:** Department of Environmental Science & Policy, University of California, Davis, Davis, 95616, USA

## Abstract

Network science has had a great impact on ecology by providing tools to characterize the structure of species interactions in communities and evaluate the effect of such network structure on community dynamics. This has been particularly the case for the study of plant-pollinator communities, which has experienced a tremendous growth with the adoption of network analyses. Here, I build on such body of research to evaluate how network structure and adaptive foraging of pollinators affect ecosystem services of plant-pollinator communities. Specifically, I quantify—using model simulations—pollen deposition in networks that exhibit structures like the ones of empirical networks (hereafter empirically connected networks) and those with higher connectance and lower nestedness than empirical networks, for scenarios where pollinators are fixed foragers and scenarios where they are adaptive foragers. I found that empirically connected networks with adaptive foraging exhibit the highest pollen deposition rate. Increased network connectance reduces pollen deposition, as increased number of interactions leads to greater conspecific pollen dilution in the absence of other mechanisms such as pollinator floral constancy. High nestedness in moderately connected networks increases the proportion of pollinators visiting only one or two plant species, which are associated with the highest quality visits. Adaptive foraging allows pollinators to quantitatively specialize on specialist plant species, which increases conspecific pollen deposition. This research advances pollination biology by elucidating how population dynamics, consumer-resource interactions (i.e., pollinators foraging on floral rewards), adaptive foraging, and network structure (i.e., nestedness and connectance) affect pollen deposition in a network context.

## Introduction

Network science has substantially contributed to ecology by providing tools to characterize the structure of species interactions within communities and assess how these structures affect community dynamics. This influence is particularly evident in the study of plant-pollinator communities, which have experienced substantial growth due to network analyses. These analyses have enabled much progress in the description of the structure of species interaction within plant-pollinator communities (Bascompte and Jordano 2007; Dormann et al. 2009) and in analyzing the effects of this network structure on the stability of plant-pollinator communities (reviewed in Valdovinos 2019). Such progress, however, has yet to focus on key dynamical and functional properties of plant-pollinator systems beyond their stability, such as quantifying and understanding the mechanisms that determine pollination services.

Pollination services provided by plant-pollinator communities sustain terrestrial biodiversity (Thompson 1994; Ollerton et al. 2011; Ollerton 2017) and food security (Garibaldi et al. 2013; Potts et al. 2016). Despite this key role, it is still unknown how the structure of plant-pollinator networks affects the pollination services these communities provide. Part of the problem is the limitations of Lotka–Volterra type models, which have been widely used to study population dynamics in mutualistic networks (e.g., Bascompte et al. 2006; Bastolla et al. 2009; reviewed Valdovinos 2019) due to their simplicity and mathematical convenience. These models depict mutualistic relationships as net positive effects between species, using a positive term in each mutualist’s growth equation that depends on the partner’s population size. However, by assuming net positive effects phenomenologically, these models cannot quantify the pollination services provided by pollinators to plants nor the effect of network structure on those pollination services. Those mechanisms are confounded together in the net positive effect of the pollinators on the plants.

A more mechanistic alternative to Lotka–Volterra type models is the consumer-resource model by Valdovinos et al. (2013, 2016, 2018). This model breaks down net effects assumed as always positive by Lotka–Volterra models into their biological mechanisms. One of its key innovations is separating plant vegetative dynamics from plant reward dynamics, allowing (1) tracking plant reward depletion, (2) assessing exploitative competition among animals visiting the same plants, and (3) incorporating adaptive foraging (behavioral responses to resource availability, Stephens and Krebs 1987; Valdovinos et al. 2010). Another key advancement most related to the study of pollination services is separating the different parts of a plant’s life cycle in processes that affect seed production and those that affect seed recruitment. Interaction with pollinators determines seed production via the quantity and quality of visits a plant receives, which are at the core of the pollination services provided by pollinators. Seed recruitment is determined by plant competition for resources other than pollinators, including shared soil resources and light, which is modeled more phenomenologically.

**Fig. 1:**
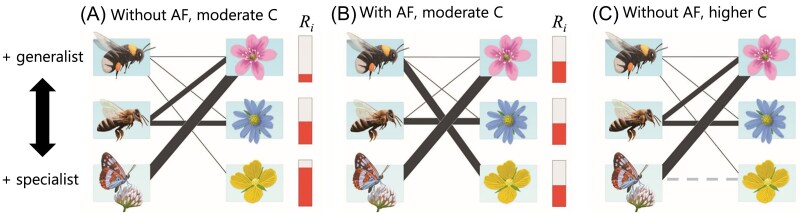
Behavior of the model and rational behind hypotheses. (A) Without adaptive foraging, pollinators assign the same fixed foraging effort to each of the plant species they visit (follow thickness of the links connecting each pollinator and plant species), which results in generalist plants receiving more and higher quality of visits and having reduced floral rewards (red bars) than specialist plants. Thus, my first hypothesis is that nestedness decreases pollen deposition rate by increasing dilution of conspecific pollen carried by generalist pollinators. Nestedness (represented in all panels) increases the fraction of interactions shared by generalist and specialist species, for both plants and pollinators. For example, all three plant species share the pollination services performed by the most generalist pollinator species, the bumblebee. (B) With adaptive foraging (AF), pollinators reassign more foraging effort to the plant species in their diet that have higher floral rewards available (specialist plant species), which results in specialist plants receiving more and higher quality of visits than without adaptive foraging. Thus, my second hypothesis is that adaptive foraging increases overall pollen deposition rate. My third hypothesis is that increasing connectance, *C*, illustrated by adding a new interaction (gray dashed line) in panel C, decreases pollen deposition rate by decreasing pollinator visit quality. For example, the added interaction in (C) results in the quality of visits of the specialist pollinator assigned to the generalist plant from *σ_ij_* = 1 to *σ_ij_* = 0.5 (see the “Methods” section).

I use the Valdovinos et al.’s dynamic model to test three hypotheses (Fig. 1) on how network structure and adaptive foraging affect pollination services, which I quantify as pollen deposition rate. First, I hypothesize that nestedness (i.e., the tendency of generalist species to interact with both specialist and generalist species and specialist species to interact with only generalist species) will decrease pollen deposition rate in networks without adaptive foraging by increasing niche overlap among plant species for pollination services of shared pollinator species (Fig. 1A). Second, I hypothesize that adaptive foraging will increase pollen deposition rates through niche partitioning between generalist and specialist species (Valdovinos et al. 2013, 2016). This mechanism will reduce conspecific pollen dilution, as generalist pollinators will focus their foraging efforts on specialist plants while specialist pollinators continue to visit generalist plants. Consequently, both generalist and specialist plants will receive higher loads of conspecific pollen and lower loads of heterspecific pollen compared to networks without adaptive foraging (Fig. 1B). Third, I hypothesize that increasing network connectance (i.e., the fraction of potential interactions that are realized) decreases pollen deposition rate. This is because higher connectance results in pollinators visiting more plant species, which—without other mechanisms in place (e.g., pollinator floral constancy)—will increase conspecific pollen dilution via increasing the hetrospecific pollen pollinators carry (Ne'eman et al. 2010; Brosi 2016). Throughout this manuscript, the terms “specialist” and “generalist” species refer to realized interactions within a network context, not to evolutionary “true” specialization.

## Methods

The Valdovinos et al’s model (Valdovinos et al. 2013, 2016; Valdovinos and Marsland 2020) defines the population dynamics of each plant (Equation 1) and pollinator species (Equation 2) of the network, as well as the dynamics of floral rewards (Equation 3) of each plant species and the foraging effort (Equation 4) that each pollinator species (per-capita) assigns to each plant species in its diet. Note that ${{\alpha }_{ij}} = 0$, if pollinator *j* does not visit plant *i*, that is, ${{\alpha }_{ij}}$ encapsulates the plant-pollinator network. Important to the objective of quantifying pollination services is that this model calculates the quantity and quality of visits received and performed by each plant and pollinator species and the resulting conspecific pollen deposition rate. Table 1 lists all the variables and parameters of the model, their definition, values, and units. Table 2 lists the biological mechanisms in the model with their assumptions. The governing equations of this model are as follows:


(1) \begin{eqnarray*}
&& \overbrace {\frac{{d{{p}_i}}}{{dt}}}^{{\mathrm{Population\ growth\ of\ plant\ sp\ }}i}\\&& \quad = \overbrace {{{\gamma }_i}{{e}_i}\mathop \sum \limits_{j \in {{A}_i}} {{\sigma }_{ij}}{{V}_{ij}}}^{{\mathrm{recruitment\ from\ animal\ pollination\ reduced\ by\ competition}}} \\&& \qquad - \overbrace {\mu _i^P{{p}_i}}^{{\mathrm{mortality loss}}}.
\end{eqnarray*}

(2) \begin{eqnarray*}
&& \overbrace {\frac{{d{{a}_j}}}{{dt}}}^{{\mathrm{Population\ growth of\ animal}}\ sp\ j}\\
&=& \overbrace {{{c}_j}\mathop \sum \limits_{i \in {{P}_j}} {{V}_{ij}}{{b}_{ij}}\frac{{{{R}_i}}}{{{{p}_i}}}}^{{\mathrm{recruitment}\ \mathrm{to}\ \mathrm{adults}\ \mathrm{from}\ \mathrm{rewards}\ \mathrm{consumption}}}\\&& \quad - \overbrace {\mu _j^A{{a}_j}}^{{\mathrm{mortality}\ \mathrm{loss}}}.
\end{eqnarray*}

(3) \begin{eqnarray*}
&& \overbrace {\frac{{d{{R}_i}}}{{dt}}}^{{\mathrm{Floral - rewards\ dynamics\ of\ plant}}\ sp\ i} = \overbrace {{{\beta }_i}{{p}_i} - {{\varphi }_i}{{R}_i}}^{{\mathrm{saturated\ production\ of\ rewards}}} \\&& \quad - \overbrace {\mathop \sum \limits_{j \in {{A}_i}} {{V}_{ij}}{{b}_{ij}}\frac{{{{R}_i}}}{{{{p}_i}}}}^{{\mathrm{consumption\ by\ pollinators}}}.
\end{eqnarray*}

(4) \begin{eqnarray*}
&&\!\!\!\!\!\!\overbrace {\frac{{d{{\alpha }_{ij}}}}{{dt}}}^{{\mathrm{Adaptive\ foraging}}}= {{G}_j}{{\alpha }_{ij}}\\&&\times\left( {\overbrace {{{c}_j}{{\tau }_{ij}}{{b}_{ij}}{{R}_i}}^{ {\it R\ \mathrm{intake\ from\ plant}}\ \mathit{ i}} - \overbrace {\mathop \sum \limits_{k \in {{P}_j}} {{\alpha }_{kj}}{{c}_j}{{\tau }_{kj}}{{b}_{kj}}{{R}_k}}^{\mathrm{average}\ \it R\ \mathrm{intake}\ \mathrm{from}\ all\ \mathit{j}^{\prime}s\ \mathrm{plants}}} \right)\!.
\end{eqnarray*}

**Table 1:** Model state variables, functions, and parameters.

Definition	Symbol	Dimension	Mean value
State variables			
Density of plant population *i*	${{p}_i}$	Individuals area^−1^	0.5*
Density of animal population *j*	${{a}_j}$	Individuals area^-1^	0.5*
Total density of floral resources of plant population *i*	${{R}_i}$	Mass area^-1^	0.5*
Foraging effort of *j* on *i*	${{\alpha }_{ij}}$	None	$\frac{1}{{\# {{P}_j}}}$ *
Functions			
Visitation rate of *j* to *i* (quantity of visits)	${{V}_{ij}} = {{\alpha }_{ij}}{{\tau }_j}{{a}_j}{{p}_i}$	Visits area^-1^ time^-1^	Variable
Quality of visits (per-capita) of *j* to *i* (per-capita)	${{\sigma }_{ij}} = \frac{{{{V}_{ij}}}}{{\mathop \sum \nolimits_{k \in {{P}_j}} {{V}_{kj}}}}$	None	Variable
Fraction of seeds *i* that recruit to adults	${{\gamma }_i} = {{g}_i}\left( {1 - \mathop \sum \limits_{l\neq i \in {{P}_j}} {{u}_l}{{p}_l} - {{w}_i}{{p}_i}} \right)$	None	Variable
Parameters			
Visitation efficiency	${{\tau }_j}$	Visits area time^-1^ individuals^-1^ individuals^-1^	1
Expected number of seeds produced by a pollination event	${{e}_i}$	Individuals visits^-1^	0.8
Per capita mortality rate of plants	$\mu _i^{( P )}$	Time^-1^	0.001
Conversion efficiency of floral resources to pollinator births	${{c}_j}$	Individuals mass^-1^	0.2
Per capita mortality rate of pollinators	$\mu _j^{( A )}$	Time^-1^	0.001
Pollinator extraction efficiency of resource in each visit	${{b}_j}$	Individuals visits^-1^	0.4
Maximum fraction of total seeds that recruit to plants	${{g}_i}$	None	0.4
Inter-specific competition coefficient of plants	${{u}_i}$	Area individuals^-1^	0.06
Intra-specific competition coefficient of plants	${{w}_i}$	Area individuals^-1^	1.2
Production rate of floral resources	${{\beta }_i}$	Mass individuals^-1^ time^-1^	0.2
Self-limitation parameter of rewards production	${{\varphi }_i}$	Time^-1^	0.04
Adaptation rate of foraging efforts of pollinators	${{G}_j}$	None	2

Values were drawn from a uniform random distribution with the specified mean and variances of 10% and 0% of means for plants’ and animals’ parameters, respectively. Parameters were taken from Valdovinos et al. (2013) and (2018). Asterisks indicate initial conditions. ${{P}_j}$ is the number of plant species that animal *j* visits.

**Table 2:** Model’s biological processes (and where they appear), their mathematical expression, and assumptions.

Biological process (Equation)	In the model	Assumption
Visitation rate (all four equations)	${{V}_{ij}} = {{\alpha }_{ij}}{{\tau }_j}{{a}_j}{{p}_i}$	Depends on the pollinator *j*’s foraging effort assigned to plant *i* (${{\alpha }_{ij}}$), *j*’s flying efficiency (${{\tau }_j}$), and the densities of plant (${{p}_i}$) and pollinator (${{a}_j}$) species
Pollen deposition rate by one pollinator species (Equation 1)	${{\sigma }_{ij}}{{V}_{ij}}$	Only a fraction of pollinator visits of *j* to *i* (${{V}_{ij}}$) produces pollination events, determined by the proportion of conspecific pollen carried by the pollinator (visit quality function ${{\sigma }_{ij}}$, see Table 1)
Per-plant pollen deposition rate (for results)	${{\sigma }_{ij}}\frac{{{{V}_{ij}}}}{{{{p}_i}}}$	Used for all pollen deposition results, either by summing over all pollinator species visiting plant species *i* (i.e., total per-plant pollen deposition rate; Figs. 2B-E, 5C), or summing over all the plant species a pollinator species visits (i.e., total per-plant pollen deposition rate by a pollinator species; Fig. 3)
Total pollen deposition received by a focal plant (Equation 1)	$\mathop \sum \limits_{j \in {{A}_i}} {{\sigma }_{ij}}{{V}_{ij}}$	Pollination events summed over all the pollinator species visiting plant species *i* (set ${{A}_i}$)
Total seed production (Equation 1)	${{e}_i}\mathop \sum \limits_{j \in {{A}_i}} {{\sigma }_{ij}}{{V}_{ij}}$	Only a fraction of the total pollination events become seeds, determined by the seed production efficiency of the plant species (parameter ${{e}_i}$)
Seed recruitment (Equation 1)	${{\gamma }_i}{{e}_i}\mathop \sum \limits_{j \in {{A}_i}} {{\sigma }_{ij}}{{V}_{ij}}$	Only a fraction of seeds produced recruit to adults, determined by the competition among plants (function ${{\gamma }_i}$)
Rewards consumption (Equation 2)	${{V}_{ij}}{{b}_j}\frac{{{{R}_i}}}{{{{p}_i}}}$	In each visit, pollinators consume floral rewards offered by the plant individual (${{R}_i}/{{p}_i}$) at a rate ${{b}_j}$
Recruitment to adult pollinators (Equation 2)	${{c}_j}\mathop \sum \limits_{i \in {{P}_j}} {{V}_{ij}}{{b}_j}\frac{{{{R}_i}}}{{{{p}_i}}}$	Total floral rewards consumed by the pollinator species (summed over all the plant species it visits, set ${{P}_j}$) are converted into new pollinators at a rate ${{c}_j}$
Rewards production (Equation 3)	${{\beta }_i}{{p}_i} - {{\varphi }_i}{{R}_i}$	Total floral rewards of a plant species *i* increases with plant density in a saturating manner, decelerating as rewards increase up to the maximum of ${{\beta }_i}{{p}_i}/{{\varphi }_i}$ when the rewards production stops
Adaptive foraging	Equation (4)	A pollinator increases its foraging effort to plants with more rewards, by reassigning its efforts from plants with fewer rewards
Efforts of a fixed forager (simulations)	$\frac{1}{{\# {{P}_j}}}$	Pollinators without adaptive foraging are assumed to have fixed foraging efforts across all the plants they visit equal to the inverse of its number of interactions, $\# {{P}_j}$ (number of plant species it visits)

To summarize, the population growth rate of plant species *i* is calculated by multiplying plant competition for resources other than pollination, expected seeds per pollination, and total conspecific pollen deposition from all animal species, then subtracting mortality-based population loss (Equation 1). Variable ${{\sigma }_{ij}}$ in Equation 1 represents the fraction of plant *i*’s conspecific pollen that pollinator *j* carries (Table 2). Second, the population growth rate of animal species *j* is determined by multiplying the efficiency of floral rewards-to-pollinator births by the total rewards consumed across all visits, then subtracting mortality-based population loss (Equation 2). Third, floral rewards dynamics of plant *i* is calculated by subtracting rewards self-limitation from rewards production, then subtracting rewards consumption by pollinators visiting plant *i* (Equation 3). Finally, adaptive foraging is modeled as changes in foraging effort that pollinator *j* assigns to plant *i*, where the difference between reward intake from plant *i* and the average intake from all plants in the pollinator’s diet determines whether *j*’s foraging effort will increase or decrease, while the adaptation rate will affect the magnitude of such change (Equation 4). All foraging efforts of a pollinator species across all plant species in its diet sum to 1.

**Fig. 2:**
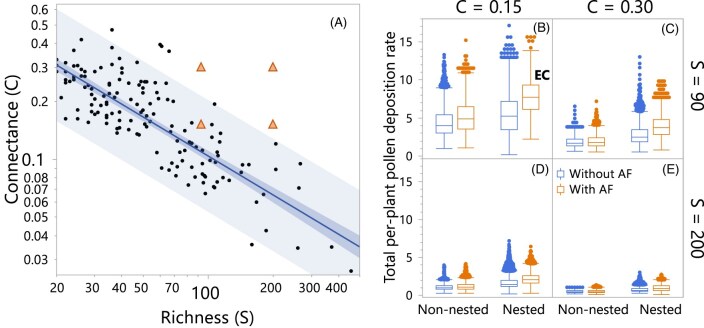
Effects of network structure and adaptive foraging on pollen deposition rate. Panel A shows connectance (*C*) vs. species richness (*S*) of 172 empirical plant-pollinator networks obtained from https://www.web-of-life.es/ that contain between 15 and 500 species. Dots represent the empirical networks. The blue solid line and darker shaded area represent the regression between *C* and *S* and its confidence interval, respectively. The lighter shaded area is the 95% prediction interval. The orange triangles represent the *C* and *S* at where the algorithm-generated networks were centered, that is *S* = 90 and *C* = 0.15, *S* = 90 and *C* = 0.30, *S* = 200 and *C* = 0.15, and *S* = 200 and *C* = 0.30. Generated networks are considered empirically connected (EC) if their *S* and *C* values land inside the 95% prediction interval and are nested. The other four panels show the pollen deposition rate summed over all visits received by plant species on a per-capita basis (see Table 2) for networks centered at *S* = 90 (B, C) and *S* = 200 (C, D) with connectance centered at *C* = 0.15 (B, D) and *C* = 0.30 (C, E), each at two levels of nestedness (non-nested and nested), totaling 800 networks, without (blue or left of each pair) and with (orange or right of each pair) adaptive foraging (AF). Boxes indicate the first and third quantile, with the middle line representing the median, and error bars represent max and min values without outliers. Dots represent outliers.

I applied this model to 800 networks generated by a widely used algorithm (Thébault and Fontaine 2010) that allows manipulating the networks’ species richness, connectance, and nestedness. I generated 100 networks to have species richness (*S*) and connectance (*C*) centered at *S* = 90 and *C* = 0.15, *S* = 90 and *C* = 0.30, *S* = 200 and *C* = 0.15, and *S* = 200 and *C* = 0.30, half non-nested and the other half nested (Fig. 2A). That is, 100 networks per each of the 8 network-structure treatments (2 richness levels × 2 connectance levels × 2 nestedness levels). “Centered at” means that the networks were generated with those *S* and *C* values as target but the actual values ranged between 87 and 91 for networks labeled as *S* = 90, between 198 to 201 for networks labeled as *S* = 200, between 0.14 to 0.16 for networks labeled as *C* = 0.15, and between 0.25 and 0.35 for those labeled as *C* = 0.3. Only the 100 nested networks centered at *S* = 90 and *C* = 0.15, are empirically connected (noted by EC in Fig. 2B), that is, exhibit structures like the ones of empirical networks (i.e., orange triangle within the 95% prediction interval, which is the lighter blue shaded area in Fig. 2A). The 172 empirical plant-pollinator networks (black dots in Fig. 2A) were obtained from the Web of Life (https://www.web-of-life.es/). The other three treatments exhibit higher connectance than the empirical networks (orange triangles outside the 95% prediction interval of Fig. 2A). I ran the model for each of the 800 networks without and with adaptive foraging. Pollinators in networks without adaptive foraging assign fixed and equal foraging efforts to each of the plant species in their diet (see Table 2).

## Results

**Fig. 3:**
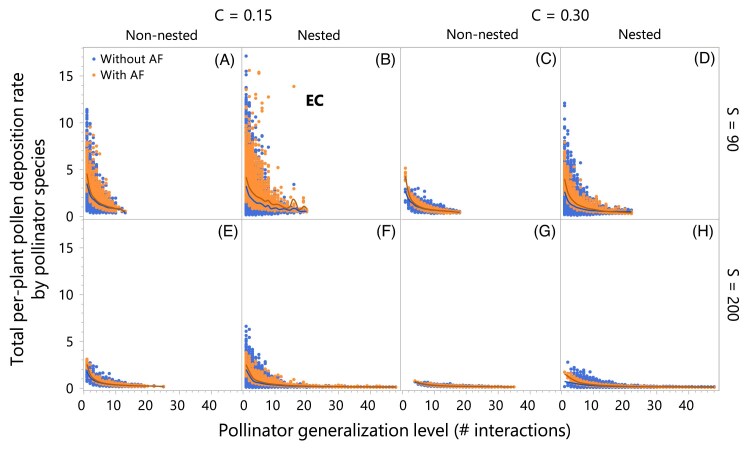
Pollinator species visiting the fewest plant species are the most efficient pollinators in terms of pollen deposition rate. Dots represent the total per-plant pollen deposition rate by each pollinator species in each of the 800 networks, without (blue) and with (orange) adaptive foraging (AF). Panels A–D show results for networks with species richness centered at *S* = 90, while panels E–H show results for networks with richness centered at *S* = 200. Panels A, B, E, F show results for networks with connectance centered at *C* = 0.15, while panels C, D, G, and H show results for networks with connectance centered at *C* = 0.3. Panels A, E, C, and G show results for non-nested networks, while panels B, F, D, H show results for nested networks. Panel B shows the empirically connected (EC) networks, which exhibit the highest pollen deposition rate by specialist pollinators. Conversely, the more overconnected the networks are with respect to what is observed in empirical networks (Fig. 2A), the fewer the specialist species, with zero specialists in non-nested networks with species richness centered at *S* = 90 and connectance at *C* = 0.3 (see Fig. 4D).

**Fig. 4:**
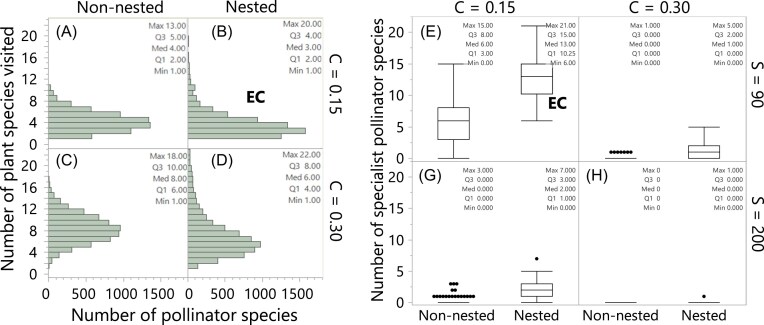
Pollinator generalization levels in simulated networks. Panels (A–D) show histograms for number of plant species each pollinator species visits across all networks with species richness centered at *S* = 90, separated by nestedness (non-nested and nested) and connectance (*C* = 0.15 and *C* = 0.3) levels. Panels (E–H) show the number of specialist pollinator species (i.e., those visiting only one plant species) in all 8 network-structure treatments, separated by species richness (*S* = 90 and *S* = 200), connectance (*C* = 0.15 and *C* = 0.3), and nestedness (non-nested and nested) levels. Note that the number of specialist species from panels A and B (i.e., visiting only 1 plant species) are both plotted in panel E, while the number of specialist pollinators from panels C and D are plotted in panel F. The histograms of networks with species richness centered at *S* = 200 are not shown due to space limitations, but they look as normal distributions like panel C. Text inside every panel indicates the maximum (Max), median (Med), minimum (Min), and the first (Q1) and third (Q3) quartiles.

Among all 8 network-structure treatments of 100 networks each, the empirically connected (i.e., nested with *S* = 90 and *C* = 0.15; EC in Fig. 2B) exhibit the highest per-capita pollen deposition rate. Increasing connectance beyond what is observed in empirical networks reduces pollen deposition rate in all richness and nestedness levels consistently with my third hypothesis (Fig. 1C). The mechanism explaining the negative effect of increased connectance on pollen deposition rate rests in the number of specialist pollinator species in a network. Pollinators visiting fewer plant species are the most efficient in depositing conspecific pollen (Fig. 3) with specialist pollinators (those visiting only one plant species) being the most efficient of all pollinators. The more overconnected the networks are with respect to what is observed in empirical networks (Fig. 2A), the fewer the specialist species in the network (Figs. 3, 4E–H). For example, the most overconnected treatments (i.e., the 200 non-nested and nested networks centered at *S* = 200 and *C* = 0.3) have zero specialist pollinator species with the only exception of one nested network that has one specialist pollinator species.

Contrary to my first hypothesis (Fig. 1A), nestedness increases pollen deposition rate. This positive effect of nestedness is explained by the relationship between nestedness and the distribution of pollinators’ generalization levels. More nested networks exhibit more specialist pollinator species (i.e., those visiting only one or a few plant species) and a few very generalized species visiting most of the plant species in the network (Fig. 4B, D). Fig. 4A–D show histograms for number of plant species each pollinator species visits (i.e., level of generalization) for networks with species richness centered at *S* = 90. The level of pollinator generalization of the empirically connected networks (nested with *C* = 0.15; Fig. 4B) is the most skewed to specialist species, with a median of 3 plant species visited, more than 1000 pollinator species visiting 1 plant species, and a long tail of a few pollinator species visiting a maximum of 22 plant species (note that the 400 networks with 90 species have a median of 23 plant species with minimum and maximum of 20 and 36, respectively). In contrast, non-nested networks with the same connectance (*C* = 0.15) and species richness (*S* = 90) exhibit a minimal tail with the most generalist pollinators visiting up to 13 plant species (Fig. 4A). More importantly for increasing pollen deposition rate in the model, nestedness increases the proportion of specialist pollinators that visit only one plant species (compare the number of specialist pollinators in non-nested and nested networks in Fig. 4E), which are the pollinators performing the highest pollen deposition rate (Fig. 3).

**Fig. 5:**
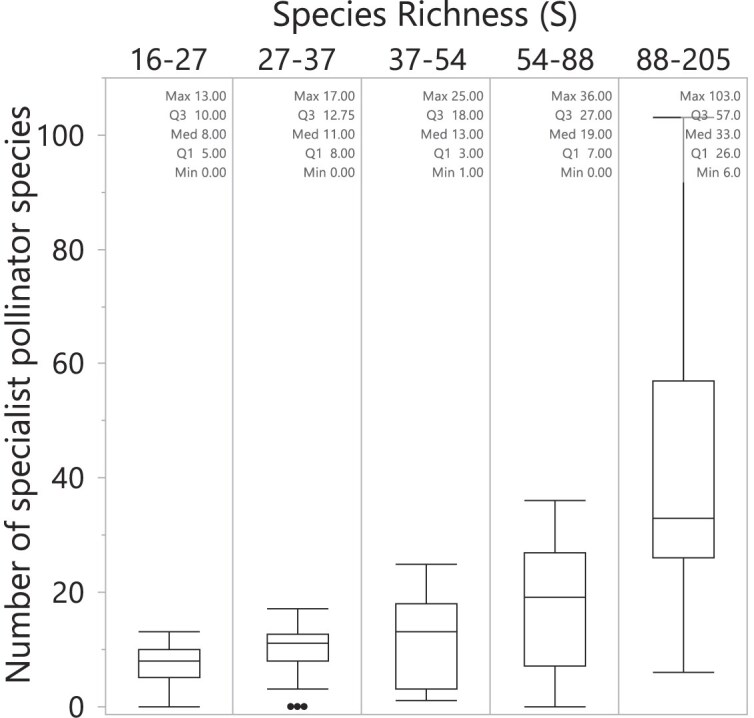
Number of specialist pollinator species in empirical networks. Empirical networks have more specialist pollinator species than the simulated networks, number that increases with species richness. At the species richness range comparable with the empirically connected networks (i.e., *S* = 88–205), the empirical networks have a median of 33 pollinator species (with minimum and maximum of 6 and 103, respectively). Boxes indicate the first and third quantile with middle line representing the median, and error bars represent max and min values without outliers. Dots represent outliers.

As mentioned above, the empirically connected (EC, Fig. 4E) have the largest number of specialist pollinators among the simulated networks, with a median of 13 (minimum and maximum of 6 and 21, respectively). Empirical networks, however, exhibit even higher number of specialist pollinator species (Fig. 5) than the EC networks. At the species richness range comparable with the simulated networks (i.e., *S* = 88–205 in Fig. 5), the empirical networks have a median of 33 pollinator species (with minimum and maximum of 6 and 103, respectively). Considering that the number of specialist pollinator species increases with species richness (Fig. 5), even empirical networks with fewer species than the EC networks (see *S* = 54–88) have more specialist pollinator species, with a median of 19 (minimum of 0 and maximum of 36).

**Fig. 6:**
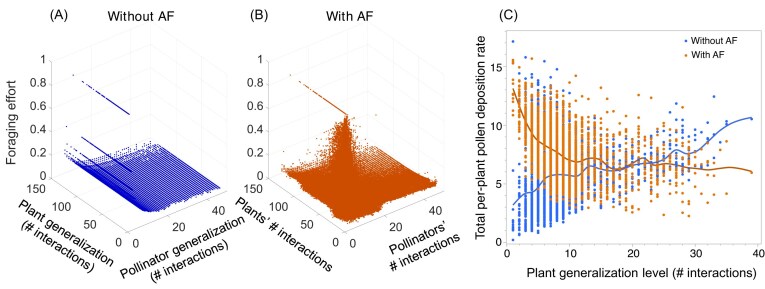
Adaptive pollinators quantitatively specialize on specialist plant species (low generalization level), which increases pollen deposition for specialist plants and decreases pollen deposition for generalist plants. Foraging efforts that pollinators assign to plants with respect to the pollinator generalization level (i.e., number of plants they visit or their number of interactions with plant species) and plant species interacting in networks without (A) and with (B) adaptive foraging (AF). (A) Without AF, all pollinator species visiting the same number of plant species have the same fixed foraging effort of ${{\alpha }_{ij}} = 1/\# {{P}_j}$, where $\# {{P}_j}$ is the number of plant species pollinator *j* visits (i.e., # interactions). (B) With AF, pollinators quantitatively reassign their foraging efforts from generalist to specialist plants. (C) Total per-plant pollen deposition rate with respect to plant generalization level without (blue) and with (orange) adaptive foraging (AF). Note that the positive relationship between pollen deposition rate and plant generalization level without AF is reversed with AF. That is, the preference of adaptive pollinators for specialist plants results in specialist plant species having a higher per-plant pollen deposition rate than generalist plant species in networks with AF, while the inverse happens in networks without AF. The line of points at level of generalization 1 in both Figs. 6B and C, correspond to specialist pollinator species, whose foraging effort and quality of visit stay at its maximum of 1, which is assigned to the only plant species in their diet. Panel B shows axis labels “Plants’ # interactions” and “Animals’ # interactions” as more specific abbreviations of plant generalization and pollinator generalization, respectively, given space limitations.

Confirming my second hypothesis (Fig. 1B), adaptive foraging increases pollen deposition rate but only slightly and mostly in EC networks (Fig. 2B). A clearer result of adaptive foraging on pollen deposition, however, is in inverting its relationship with plant generalization from positive to negative (Fig. 6A). That is, adding adaptive foraging to the network dynamics results in specialist plants experiencing higher pollen deposition rates than generalist plants, while generalist plants exhibit higher pollen deposition rates than specialists in networks with fixed foragers. Fig. 6B shows that in networks without AF, pollinators assign the same foraging efforts (${{\alpha }_{ij}} = 1/\# P_j^{}$) to each of its plant species regardless of pollinator species visiting each plant species. Fig. 6C shows that pollinators with adaptive foraging reassign their foraging efforts to the most specialist plant species in their diet (i.e., visited by fewer pollinator species or only by them). Thus, the positive effect of adaptive foraging on pollen deposition of specialist plants occurs due to pollinators reassigning most of their foraging effort from the generalist to the specialist plant species in their diet (compare Figs 6B and C).

## Discussion

Among the eight network-structure treatments, empirically connected networks exhibit the highest per-capita pollen deposition rate. Contrary to my first hypothesis, nested networks exhibited higher pollen deposition rates than non-nested networks. Adaptive foraging slightly increases pollen deposition rates, supporting my third hypothesis, and reverses the positive relationship between pollen deposition rate and plant generalization levels. Finally, increasing connectance beyond the levels observed in empirical networks reduces pollen deposition rates across all levels of species richness and nestedness, consistent with my third hypothesis.

The effect of network structure—specifically connectance and nestedness—on pollen deposition rates is driven by the proportion of specialist pollinators within the network. Networks with a higher proportion of specialist pollinator species exhibit greater pollen deposition rates. The simulated networks that were moderately connected and highly nested (i.e., the empirically connected networks) exhibited the highest pollen deposition rates among all simulated networks due to having the highest proportion of specialist pollinator species. Indeed, the highly connected and species rich communities lacking specialists exhibited the lowest per-plant pollen deposition of all treatments. These findings suggest a critical role of specialist pollinators in maintaining ecosystem functionality: their loss may substantially decrease pollination effectiveness in an ecosystem, even when overall species richness and interaction numbers remain stable.

To evaluate whether the number of specialist pollinators in my simulated networks aligns with empirical data, I compared it to empirical networks and found that the latter contained even higher proportions of specialist pollinators. However, the true existence of such a high number of specialist pollinator species in empirical networks has been questioned (Waser et al. 1996; Brosi 2016; but see Johnson and Steiner 2000), as it may result from insufficient sampling effort rather than reflecting true ecological patterns (Blüthgen et al. 2008; Blüthgen 2010). This questioning has prompted numerous studies investigating the effects of sampling effort on network structure, both through field studies (Nielsen and Bascompte 2007; Hegland et al. 2010; Chacoff et al. 2012) and through models generating network structures (Blüthgen et al. 2008; Blüthgen 2010; Fründ et al. 2016).

These studies have consistently shown that incomplete sampling strongly underestimates the number of interactions while simultaneously overestimating the degree of specialization. However, for the present results, pollinator specialization does not need to occur at the species level for its positive effects on pollen deposition rates to hold. Instead, specialization can occur at the individual level through flower constancy (Chittka et al. 1999) or fidelity (Brosi 2016), or as temporary, realized specialization at the population level. Flower constancy (or fidelity more broadly) is the tendency of pollinators to visit flowers of the same species during a foraging bout despite the presence of other available floral resources. This behavior allows individual pollinators to act as specialists during their foraging bouts, even if the species as a whole interacts with multiple plant species. By focusing on a single plant species throughout a foraging bout, individual pollinators increase the efficiency of pollen transfer and deposition (Chittka et al. 1999; Ne'eman et al. 2010; Brosi 2016), producing results similar to those presented here.

Another mechanism that could produce results similar to those presented here is realized specialization. This term refers to pollinator species that are observed visiting only one plant species during a specific sampling period, even though they may interact with other plant species at different times or under varying conditions. These pollinators are not true specialists in an evolutionary sense but instead exhibit flexibility in their interactions, often through turnover or rewiring over time (Brosi 2016; CaraDonna et al. 2017; Glaum et al. 2021). To summarize, any mechanism of specialization—whether temporary, individual-level, population-level, or species-level—suffice for the present results of increased pollen deposition to hold.

Beyond the number of specialist pollinators, higher connectance increases the number of plant species each pollinator interacts with. This broader range of interactions per pollinator species can lead to heterospecific pollen transfer (Arceo-Gómez et al. 2020) and dilution in conspecific pollen on pollinators’ bodies. Nestedness, however, counteracts these effects by creating generalization levels skewed to more specialist plant and pollinator species. In highly nested networks, the distribution of generalization levels follows a long-tail pattern (Payrató-Borras et al. 2019), where most species have few interactions, and a small number of species dominate most interactions within the network. Therefore, nested networks will always have many pollinator species with one or few realized interactions, which will be effective pollinators in terms of pollen deposition.

Adaptive foraging further enhances pollen deposition rates in moderately connected and highly nested networks by allowing pollinators to allocate greater per-capita foraging efforts to specialist plants. Specialist plants, therefore, benefit from receiving higher-quality visits from generalist pollinators that focus their efforts on specialist plants and primarily carry their conspecific pollen (Valdovinos et al. 2013, 2016; Valdovinos and Marsland 2020). This occurs because specialist plants in the model tend to have more available rewards, as they are less frequently visited compared to generalist plants, which experience higher visitation rates (Valdovinos et al. 2013, 2016; Valdovinos and Marsland 2020).

In conclusion, this study demonstrates that the structure of plant-pollinator networks and the adaptive foraging behavior of pollinators can play critical roles in determining pollen deposition rates in plant-pollinator communities. Empirically connected networks with moderate connectance and high nestedness exhibit the highest pollen deposition rates, highlighting the importance of network structure in facilitating effective pollination. While increased connectance can dilute conspecific pollen through heterospecific interactions, nestedness counteracts this effect by creating a distribution of generalization levels that promotes specialization, even if only temporarily or at the individual level. Adaptive foraging further enhances pollen deposition by enabling pollinators to allocate more effort to specialist plants, thereby improving resource partitioning and coexistence between specialist and generalist species.

These findings emphasize the interplay between network structure, pollinator behavior, and ecosystem function, advancing our understanding of how plant-pollinator communities sustain critical ecological processes. Future research should more explicitly evaluate the interplay between individual pollinator specialization and network structure, which can be studied both empirically and by using mathematical models. By integrating network theory, behavioral ecology, and ecosystem dynamics, this work provides a framework for understanding the complex relationships that underpin ecosystem services such as pollen deposition.

## Data Availability

The code used to produce all results of this work is publicly available at the GitHub repository https://github.com/Valdovinos-Lab/PollenDeposition.

## Notes

“From the symposium“Pollinator-plant interactions in a changing landscape: embracing integrative approaches across scales” presented at the annual meeting of the Society for Integrative and Comparative Biology, January 3–7th, 2025.
